# Genetic Drift of HIV Populations in Culture

**DOI:** 10.1371/journal.pgen.1000431

**Published:** 2009-03-20

**Authors:** Yegor Voronin, Sarah Holte, Julie Overbaugh, Michael Emerman

**Affiliations:** 1 Human Biology Division, Fred Hutchinson Cancer Research Center, Seattle, Washington, United States of America; 2 Public Health Sciences Division, Fred Hutchinson Cancer Research Center, Seattle, Washington, United States of America; University of Oxford, United Kingdom

## Abstract

Populations of Human Immunodeficiency Virus type 1 (HIV-1) undergo a surprisingly large amount of genetic drift in infected patients despite very large population sizes, which are predicted to be mostly deterministic. Several models have been proposed to explain this phenomenon, but all of them implicitly assume that the process of virus replication itself does not contribute to genetic drift. We developed an assay to measure the amount of genetic drift for HIV populations replicating in cell culture. The assay relies on creation of HIV populations of known size and measurements of variation in frequency of a neutral allele. Using this assay, we show that HIV undergoes approximately ten times more genetic drift than would be expected from its population size, which we defined as the number of infected cells in the culture. We showed that a large portion of the increase in genetic drift is due to non-synchronous infection of target cells. When infections are synchronized, genetic drift for the virus is only 3-fold higher than expected from its population size. Thus, the stochastic nature of biological processes involved in viral replication contributes to increased genetic drift in HIV populations. We propose that appreciation of these effects will allow better understanding of the evolutionary forces acting on HIV in infected patients.

## Author Summary

Genetic drift can be a strong evolutionary force, especially in small populations. Studies of HIV evolution within a single infected patient suggest that genetic drift plays an important role in the evolution of the virus, despite the large size of the viral population. The factors responsible for the high genetic drift are not known, but several models have been proposed to explain the phenomenon; all of them assume that the viral population is ideal. We measured the amount of genetic drift in HIV populations, replicating in the controlled environment of cell culture. We found that HIV populations exhibit approximately 10-fold more genetic drift than would be expected for an ideal population of similar size. Non-synchronous timing of infection is partially responsible for the increase, but other unidentified factors also contribute. While the increase in genetic drift observed for HIV in culture is not sufficient to explain the several orders of magnitude increase in intra-patient genetic drift, it provides strong experimental evidence for the intrinsic stochasticity of the HIV replication cycle. Understanding the sources of genetic drift is necessary to better understand the evolutionary forces that act upon HIV *in vivo*.

## Introduction

Genetic drift is defined as stochastic fluctuations in frequencies of alleles in a population. Generally, large populations are less stochastic and undergo less genetic drift than smaller populations. While viruses exhibit very large population sizes, suggesting that the genetic processes in these populations are mostly deterministic, it has been recently appreciated that genetic drift is an important factor in virus evolution. For example, plant viruses undergo severe bottleneck events both when spreading from one plant to another and within an individual infected plant, which leads to frequent founder effects in their populations [1],[2],[3],[4],[5],[6]. Significant contribution of genetic drift has also been proposed for evolution of animal and human viruses, such as norovirus [7], measles [8], hepatitis B virus [9], coronavirus [10], Dengue virus [11], rabies virus [12], and hantavirus [13]. However, accurate determination of the role of genetic drift in evolution of animal viruses is complicated, because genetic drift, a stochastic process, is hard to discern from antigenic drift, which is a selection-driven process associated with individual differences in immune responses of infected hosts. Nevertheless, studies aimed at separating the role of the immune response still find a significant influence of genetic drift in evolution of some animal viruses [14],[15],[16].

The processes that cause genetic drift in extremely large viral populations have not been thoroughly explored, but they include events that occur during both transmission of the virus from one organism to another and viral replication within a single organism (intra-host). Studies of wheat streak mosaic virus suggest that both mechanisms can contribute significantly to viral evolution [1],[2], but whether this conclusion applies to animal viruses is not clear. The majority of the work on genetic drift in animal viruses has focused on large scale viral populations, comparing viruses either in geographically isolated regions or in consecutive epidemics and, therefore, does not distinguish between the intra- and inter-host genetic drift.

The animal virus for which intra-host genetic drift has been extensively studied is human immunodeficiency virus type 1 (HIV-1). Multiple studies observed that genetic drift of HIV-1 within an infected individual is several orders of magnitude larger than would be predicted from the total number of infected cells in the body [17],[18],[19],[20],[21]. Several models have been proposed to explain the observed high genetic drift, including multiple selective sweeps [22], metapopulation structure [23],[24] and rare but severe population bottlenecks [25]. All of these models, however, implicitly assume that viral population replicating under homogenous well-mixed conditions should behave as an ideal population. Ideal populations are expected to undergo a certain amount of genetic drift, but real viral populations are also influenced by to the stochastic nature of the biological processes involved in viral replication. Therefore, real viral populations can be expected to have an excess of genetic drift, even under “close-to-ideal” conditions. The degree to which viral replication process contributes to genetic drift in viral populations is the main interest of our study.

Here, we studied genetic drift in HIV populations replicating under the most basic conditions of cell culture. We developed a system that can be used to accurately measure genetic drift occurring in HIV populations. Using this system we show that genetic drift in HIV populations in culture is approximately tenfold higher than expected for an ideal population. Because the increase in HIV genetic drift observed in culture is due to replication process itself, it should also be present *in vivo* and, therefore, may partially explain the high genetic drift observed in HIV populations in infected people.

## Results

### Measuring Genetic Drift in HIV Populations in Culture

**Figure 1:**
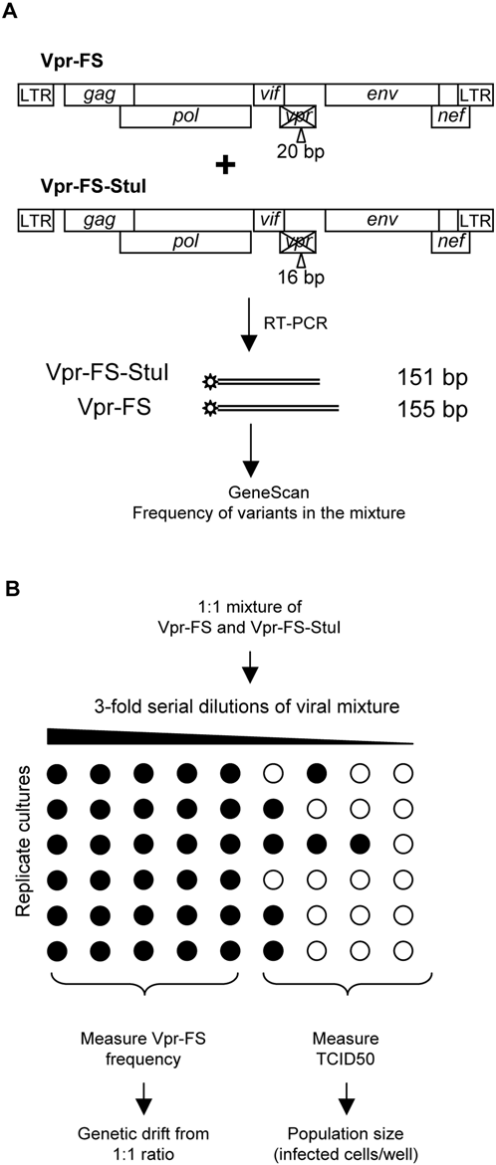
A. Difference in length of the insertion in *vpr* gene of Vpr-FS and Vpr-FS-StuI clones allows measurements of their relative abundance in mixtures. Fragments of genomes containing insertions are amplified in RT-PCR reactions using fluorescently labeled primers. Relative abundance of products of different length can be quantitated from fluorescence intensity of the corresponding bands in GeneScan assay. B. Scheme of the experimental approach used to correlate the number of infected cells to the amount of genetic drift. Viral variants Vpr-FS and Vpr-FS-StuI are mixed in 1∶1 ratio. The mixture is serially diluted and used to infect multiple replicates of cell cultures (shown 6 replicates for each dilution). Cultures are scored as positive (black circles) or negative (white circles) for HIV infection. Cell-free virus is collected from virus-positive wells and the frequency of virions of each variant is measured by the GeneScan assay. The data is used to calculate the amount of genetic drift for each dilution. Dilutions containing positive and negative wells are used to calculate TCID50, which provides the measure of viral population size at the start of the experiment.

Our approach to investigating the impact of genetic drift on HIV was to create viral populations of known size and monitor the degree of variation in the frequency of a neutral allele in these populations. An HIV population carrying a neutral allele at 50% frequency was created by mixing two replication-competent variants of HIV, Vpr-FS and Vpr-FS-StuI (Figure 1A). Both variants carry a frameshift insertion in the *vpr* gene, resulting in non-functional Vpr protein, which is not necessary for viral replication in cell culture. The insertions in the two variants differ in length by 4 bp, which allows accurate measurement of the frequency of each variant in viral mixtures by the PCR-based GeneScan assay (see Materials and Methods). Neither Vpr-FS nor Vpr-FS-StuI has an advantage for replication in culture, i.e. the variants are selectively neutral (data not shown). Thus, by mixing these two variants in a 1∶1 ratio we created a population of HIV with a known neutral allele present at a 50% frequency.

The experimental scheme we used to determine the relationship between population size and genetic drift is shown in Figure 1B. Serial 3-fold dilutions of a 1∶1 viral mixture of neutral variants were prepared and used to infect multiple independent cultures of target cells to create HIV populations of different sizes. All cultures were maintained for 5–14 days until most of the cells in virus-positive cultures were infected (see Materials and Methods). At that point, the cell-free virus from virus-positive wells was collected and analyzed by the GeneScan assay to determine the frequency of the two alleles in each of the replicate cultures. The observed Vpr-FS and Vpr-FS-StuI frequencies in each set of replicates were used to calculate the average variance of the observed frequency from the expected 50%. We used this variance as a measure of genetic drift. In parallel, the size of viral population at the beginning of each experiment was calculated by measuring tissue culture infectious dose 50% (TCID50), which the amount of virus that infects 50% of the wells at a given dilution (see figure legend to Figure 1B). In several experiments, an infectious center assay was used to confirm the estimated number of infected cells at higher dilutions and the results were always within twofold from predictions based on multiplicity of infection (data not shown).

**Figure 2:**
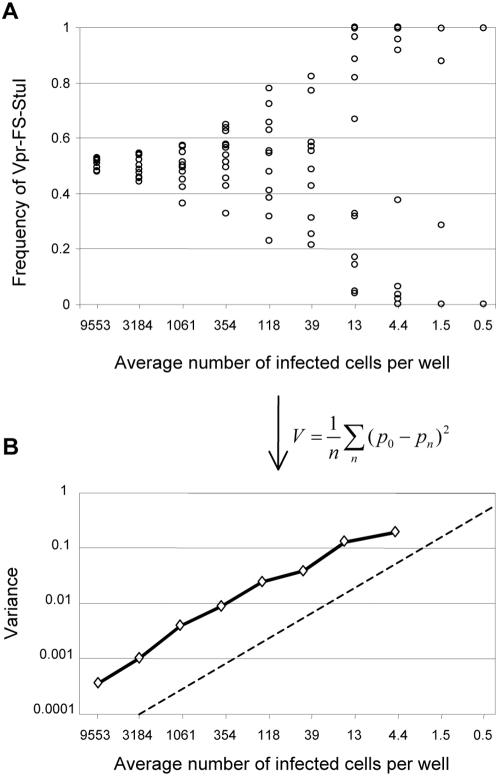
Representative results of an experiment measuring genetic drift in HIV populations. A. The frequency of Vpr-FS in replicate cultures. Each point represents one of 12 independent cultures done for each dilution. B. Variance in frequency of Vpr-FS-StuI was calculated from data shown in panel A. Each point corresponds to average variance in frequency of Vpr-FS-StuI from initial 50%, adjusted for contribution from assay variability (see Materials and Methods). The expected variance for an ideal population is shown as straight dashed line.

For illustration of the technique, representative data obtained in one genetic drift measurement experiment are shown in Figure 2. Twelve independent cultures of C8166 cells were infected for each dilution of the viral mixture. As expected, at the dilution with the highest amount of virus (9553 infected cells per well) the frequency of Vpr-FS-StuI was very close to 50% (Figure 2A). When the amount of virus used for infection was decreased, Vpr-FS-StuI frequency showed wider variation. Because the same 1∶1 viral mixture was used for all dilutions, the variation in the frequencies of the two neutral variants must have been caused by genetic drift. At the lowest viral dilution (less than one infected cell per well), only a single variant was observed in each of the five infected cultures; 7 of the cultures were not infected at this dilution. It should be noted that Vpr-FS-StuI frequency was equally likely to increase or decrease relative to the starting 50%, which confirmed that Vpr-FS and Vpr-FS-StuI have identical replicative fitness.

These data were used to calculate the variance in frequency of Vpr-FS-StuI in replicate cultures for each dilution (Figure 2B). We used this variance as a measure of the amount of genetic drift in HIV populations. As expected, the variance was lowest (0.00045) for the largest population size, indicating that genetic drift was low in these cultures. Variance increased as the population size decreased, demonstrating the predicted reciprocal relationship between the population size and genetic drift. The variance was not calculated for two dilutions with the lowest population size (1.5 and 0.5), because they contained non-infected wells. Assay variance (see Materials and Methods) in this experiment was 0.000086, i.e. was less than 20% of the lowest measured total variance (data not shown).

To better understand the sources of genetic drift in our experiments, we compared the observed variance to the variance expected to occur due to genetic drift in an ideal population. Probability theory predicts that, in a single generation, an ideal population of *N* individuals should undergo genetic drift simply due to stochastic sampling. The variance caused by this drift, to which we refer here as V_ideal_, was calculated from the initial allele frequency *p* and viral population size *N* as
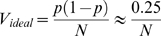
and plotted on Figure 2B as the thin dashed line. For all tested population sizes, the observed variance in frequency of neutral allele was approximately an order of magnitude higher than V_ideal_, demonstrating that, even under relatively homogenous conditions of cell culture, viral populations do not behave as ideal populations.

#### Genetic drift under a variety of culture conditions

**Figure 3:**
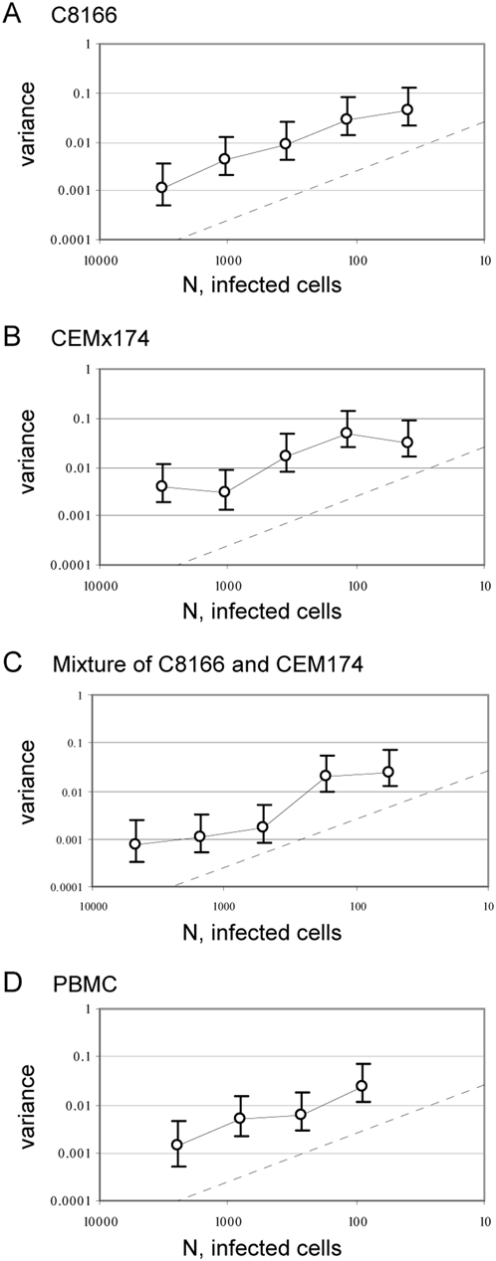
Relationship between virus population size and genetic drift in a variety of cell cultures. A. In C8166 cells. B. In CEMx174 cells. C. In 1∶1 mixture of C8166 and CEMx174 cells. D. In PBMCs. Each point corresponds to average variance in frequency of Vpr-FS-StuI from initial 50% (see Materials and Methods). Error bars are 95% confidence intervals. Population size N is defined as the average number of infected cells in each culture at the beginning of the experiment. The expected variance for an ideal population is shown as straight dashed line.

The measurement of genetic drift in HIV populations infecting C8166 cells as shown in Figure 2 was repeated a total of five times with different viral dilutions. The variance calculated from the combined data set was used as a baseline to which we compared the variance observed for other infection conditions. Additionally, statistical analysis was performed to calculate 95% confidence intervals for the estimated variance at each population size, which showed that for viral populations infecting C8166 cells, the estimated variance, to which we refer here as V_total_, was significantly higher than V_ideal_ for all population sizes (Figure 3A).

We then tested whether properties of infected cells affected the magnitude of genetic drift in HIV populations. Thus, we measured genetic drift in viral populations infecting another cell line, CEMx174. These cells differ from C8166 cells morphologically, require the presence of DEAE-Dextran for robust HIV infection, and produce smaller amounts of virus after infection than C8166 cells (data not shown). The observed variance V_total_ of Vpr-FS-StuI frequency in HIV populations infecting CEMx174 cells was also significantly higher than V_ideal_ (Figure 3B), suggesting that higher than expected genetic drift is is a common property of the virus replication process.

To test whether the variability in properties of target cells is important for genetic drift, we infected a 1∶1 mixture of the two cell types, CEMx174 and C8166. The results, shown in Figure 3C, demonstrated that the amount of genetic drift under these conditions was similar to the amount observed in infections of cultures containing either C8166 or CEMx174 cells alone.

Next we measured genetic drift in HIV populations infecting activated primary blood mononuclear cells (PBMCs). The results were similar to those obtained with immortalized cell lines and showed that genetic drift in HIV populations in primary cells was also higher than expected for an ideal population (Figure 3D), supporting our hypothesis that increased genetic drift is a consistent feature of the virus replication process, independent of target cells.

Our analysis of data from different culture conditions shows that V_total_ under all tested conditions was approximately 10-fold higher than V_ideal_ and varied from 10.1-fold to 22.7-fold (Table 1). We compared V_total_ observed under different conditions to V_total_ in C8166 infection. Interestingly, infections of CEMx174 cells resulted in statistically higher genetic drift than infections of C8166 cells (95% confidence intervals do not include 1, see Materials and Methods for statistical analysis details). However, the estimate of variance for CEMx174 cells had large standard error and the variances for the two conditions differed by only 2-fold, showing that our general estimate of one order of magnitude difference between V_ideal_ and V_total_ applies to both target cells.

**Table 1:** Genetic drift under a variety of culture conditions.

Data figure	Target cells	Condition	Fold increase of V_total_ over V_ideal_ (±SE) 1	Fold change in V_total_ compared to V_total_ in standard C8166 infection (CI)^4^
3A	C8166	Standard	10.5 (±4.5)	1 (NA)
3B	CEMx174	Standard	22.7 (±13.9)	1.44 (1.25, 1.66)^2^
3C	C8166 and CEMx174	Mixture of two cell types	10.1 (±4.2)	1.00 (0.88, 1.13)
3D	PBMCs	Standard	11.7 (±4.2)	1.08 (0.89, 1.33)
4A	C8166	Virus pre-bound to Raji-DC-SIGN	4.5 (±1.0)	0.69 (0.59, 0.82)^3^
4B	C8166	Mixture of C8166 and Raji-DC-SIGN	8.8 (±5.4)	0.95 (0.82, 1.11)
4C	C8166	Synchronized	2.8 (±0.6)	0.52 (0.44, 0.61)^3^
4D	C8166	Synchronized, single cycle	2.7 (±1.0)	0.60 (0.51, 0.70)^3^

1 Fold increase was averaged for all population sizes in all experiments. SE is standard error.

2 Variance is significantly greater than the variance of the standard C8166 condition.

3 Variance is significantly less than the variance of the standard C8166 condition.

4 Adjusted for differences in population sizes. CI is 95% confidence intervals.

Genetic drift in infections of 1∶1 mixture of C8166 and CEMx174 cells was not statistically different from genetic drift in C8166 infections, suggesting that properties of C8166 cells (higher infectivity and higher virus production) dominated in the mixed population. Genetic drift in infections of PBMCs was similar to drift observed in C8166 cells (Table 1).

### Synchronization of Infection Reduces Genetic Drift

**Figure 4:**
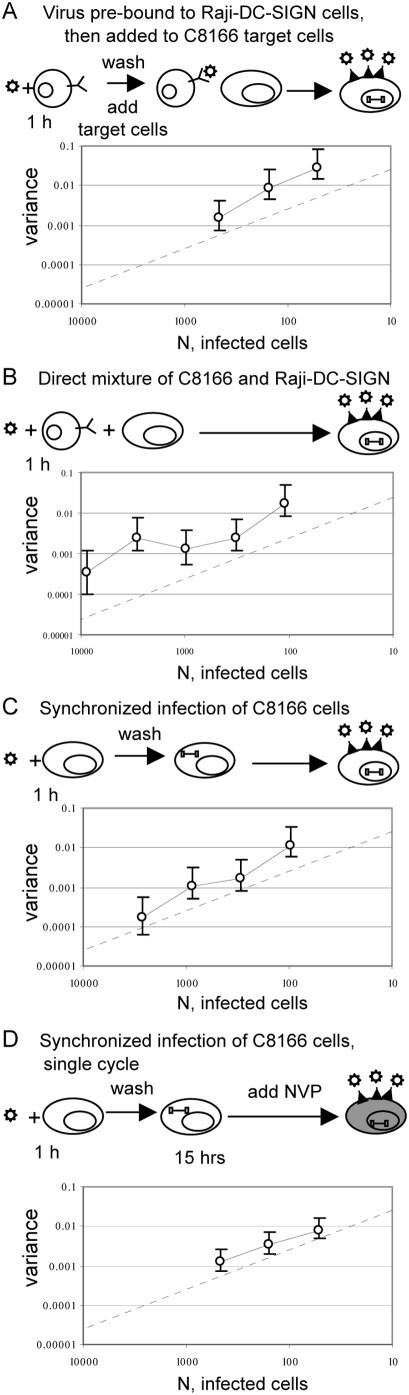
Synchronization of infection reduces genetic drift. A. Virus mixture was first incubated with Raji-DC-SIGN cells. After one hour the cells were washed to remove unbound virus and mixed with C8166 target cells. B. Raji-DC-SIGN cells were mixed with the target C8166 cells and infected. C. Infections were synchronized. Virus mixture was incubated with target cells for 1 hour, after which the cells were washed to remove unbound virus and resuspended in fresh media. D. Infection was synchronized as in panel C. After 15 hours viral replication was blocked by addition of nevirapine. Cell-free virus was collected 48 hours later, cells were washed to remove nevirapine and resuspended in fresh media to allow viral replication and TCID50 measurement. Each point corresponds to average variance in frequency of Vpr-FS-StuI from initial 50%. Population size N is defined as the average number of infected cells in each culture at the beginning of the experiment. Error bars are 95% confidence intervals. The expected variance for an ideal population is shown as straight dashed line.

We asked whether amount of genetic drift in HIV populations can be influenced by culture conditions. Thus, we measured Vpr-FS-StuI variance in cultures where the virus mixture was bound to the Raji-DC-SIGN cells prior to the addition of target C8166 cells. In order to do this, we used Raji-DC-SIGN cells, which cannot be infected by wild type HIV, but can bind the virus through the DC-SIGN molecule on their surface and enhance its infectivity by presenting the virus to the surfaces of uninfected cells [26],[27]. Raji-DC-SIGN cells were incubated with virus for 1 h and then washed three times with media to remove all unbound virus. The cells were then mixed with the target C8166 cells to mediate infection *in trans*. V_total_ under these conditions was only 4.5 fold higher than V_ideal_ (Figure 4A), a statistically significant reduction from the 10.5 ratio of V_total_ to V_ideal_ observed in direct C8166 infections (Table 1). In order to test whether genetic drift was affected by pre-bound state of the virus or the simple presence of Raji-DC-SIGN cells in culture, we mixed C8166 cells with Raji-DC-SIGN cells prior to addition of the virus (Figure 4B). Because the simple presence of Raji-DC-SIGN in culture did not affect genetic drift of HIV, as evidenced by 9.5-fold increase of V_total_ over V_ideal_ (Figure 4B and Table 1), this result suggested that the reduction of genetic drift was connected to the state of the virus in the beginning of infection. We hypothesized that binding of the virus to virus-presenting cells and removal of the unbound virus resulted in increased synchronization of timing of infection, which caused reduction in genetic drift.

We tested whether synchronization of infection has an effect on the amount of genetic drift in viral populations by changing experimental parameters to favor synchronized entry of virus entry into cells. Thus, virus was incubated with target cells for one hour, after which the unbound virus was removed by three washes with media. Synchronization of infection drastically reduced the amount of observed genetic drift (Figure 4C), so that V_total_ under these conditions was only 3-fold higher than V_ideal_. The increase was significantly lower than increase in non-synchronized infections of C8166 cells (Table 1). Similar results were obtained when the virus was incubated with cells for 3 hours, or when it was pre-bound to C8166 target cells at 8C, or spinoculated (data not shown). Thus, a large proportion of genetic drift in HIV populations was due to stochastic effects associated with non-synchronous infections of target cells.

#### Genetic drift over one cycle of viral replication

All our experiments above were based on the assumption that the first cycle of viral replication determined the amount of genetic drift in the population due to exponential growth of viruses in culture. To determine experimentally whether or not multiple cycles of viral replication influenced the amount of observed genetic drift, we measured frequency of Vpr-FS-StuI in virions produced during a single cycle of viral replication. The infection was synchronized by washing off non-bound virus after one hour of incubation with C8166 target cells as described above to avoid the increase in genetic drift that happens due to non-synchronous infection. The continuing rounds of viral replication were blocked by addition of antiviral drug nevirapine to the final concentration of 5 uM at 18 hr post-infection. At 72 h post-infection, cell-free virus was collected for the GeneScan analysis and nevirapine-free media was added to cells to allow viral replication and TCID50 measurement. Genetic drift under these conditions was similar to genetic drift in synchronized cultures after multiple rounds of viral replication (Figure 4D and Table 1). Formal comparison of these two conditions did not find a statistically significant difference in observed variance (data not shown). These data are consistent with the idea that the majority of genetic drift in viral populations in culture occurs during the first cycle of replication.

## Discussion

In this study we have shown that genetic drift of HIV populations existing under relatively homogeneous conditions of cell culture exceeds genetic drift expected for an ideal population by an order of magnitude. A large portion of the observed drift is due to the non-synchronous nature of infection, where a small proportion of virions gains reproductive advantage by quickly infecting their target cells. When infection is synchronized, the observed genetic drift is reduced, but is still approximately 3-fold higher than drift expected for an ideal population.

Genetic drift depends on the size of the population in question. However, the definition of an individual and, therefore, of a population size, is somewhat complicated for viruses. Individual virions do not contribute to the next generation unless they infect a target cell. The number of infected cells, therefore, is a better measure of population size, and is quite common in HIV population genetics literature [17],[18],[22]. Yet the fact that a single cell can be infected by more than one virion adds some confusion. The latter problem was avoided in our study by starting experiments at low multiplicity of infection (<0.05), which makes double-infection unlikely. We defined the size of viral population as the total number of cells infected in the culture by the virus added at the beginning of the experiment.

The reasons for the increased genetic drift in HIV populations are not entirely clear. Our work provides strong evidence for the importance of synchronization of infection for reduction of genetic drift. While we have not measured kinetics of infection, it is logical to assume that some of the infections occurred within minutes of addition of virus to cells, while others occurred hours, or even days, later. Indeed, when input virus was washed off after 1 hr during synchronization experiments, viral titers were reduced 10–100-fold as compared to non-synchronized infection (data not shown). As a result, in non-synchronized infections there exists an “early” virus population, which has a temporal advantage over the “late” viral population. “Early” population produces new virions faster and contributes to the next generation of infected cells more than the “late” population. Therefore, the small size of the “early” population defines to a large degree the amount of genetic drift observed in the total population. The potential influence of such non-discrete generations on genetic drift is a well-described phenomenon in population genetics (for review see [28]). Indeed, we found that genetic drift can be significantly reduced when infections are synchronized. Synchronization removes the “late” viral population and, therefore, the total size of viral population becomes much closer to the size of the “early” population. This results in a more synchronous production of virus particles in the second generation (the major contributor to genetic drift), which becomes discrete reducing the observed genetic drift.

Yet even in synchronized infections, genetic drift is higher in HIV populations than in an ideal population. We believe that the reasons for that lie in differences in metabolic state of target cells, their lifespan, or expression levels of positive and negative factors involved in viral replication. These and other individual differences between target cells can introduce stochastic events into the viral life cycle. For example, it has recently been proposed that positive feedback loops in transactivation of RNA synthesis by viral protein Tat lead to stochastic differences in levels of viral gene expression [29]. As a result, the viral population is randomly divided into actively replicating and latent subpopulations. In general, these and other factors may lead to a non-Poisson distribution in the number of progeny per parent and affect the amount of genetic drift in the population [30]. Interestingly, our data showed that genetic drift in a heterogeneous mixture of C8166 and CEMx174 cells was similar to genetic drift in a more homogeneous C8166 culture, which appears to contradict this prediction. However, it is possible that the differences between target cells in those experiments were not sufficient to result in an observable effect. Additional experiments are needed to establish the relevant biological differences of different target cells and the effect of those differences on genetic drift in viral populations.

All current models of HIV genetic drift implicitly assume that the process of viral replication itself is stochastic only to the degree of an ideal population of a given size [21],[22],[23],[25]. Our results show that this is not the case. Populations of HIV in cell culture undergo approximately tenfold more genetic drift than would be expected from their population sizes. This increase is not sufficient to explain the several orders of magnitude excess in genetic drift of HIV observed in patients [17],[19],[20],[21], but it provides experimental evidence for one source of genetic variation in HIV populations. Indeed, the factors that we proposed to explain the increased genetic drift of HIV in culture should play similar, or even larger, roles in HIV populations in patients. There, infections should be less synchronized than in culture and the individual differences between target cells should be larger than in cell lines or highly stimulated PBMCs that we used in our experiments. Our data suggest that the existing models which explain this excess of genetic drift through multiple selective sweeps [22], metapopulation structure [23],[24] or rare severe population bottlenecks [25] may overestimate the influence of those factors on HIV population genetics. We believe our findings will allow creation of better models describing forces acting on HIV population genetics in an infected person. Genetic drift is a powerful evolutionary force and understanding the factors contributing to it is crucial for our understanding of HIV evolution.

## Materials and Methods

### Cells and Viruses

C8166 and CEMx174 cells, expressing secreted alkaline phosphatase (SEAP), were a kind gift of Dr. R. Desrosiers [26]. Raji-DC-SIGN cells have been previously described as B-THP-1 cells [27]. All non-adherent cell lines were maintained in RPMI 1640 medium (Invitrogen) supplemented with 7% bovine growth serum, 2 mM L-glutamine, 100 U/ml penicillin and 100 µg/ml streptomycin. Human 293T cells (ATCC) were maintained in DMEM medium (Invitrogen) supplemented with 7% bovine growth serum and antibiotics. Human peripheral blood mononuclear cells (PBMCs) were isolated from healthy donors using Ficoll gradient and maintained in RPMI 1640 medium supplemented with 10% fetal bovine serum, 2 mM L-glutamine and antibiotics. PBMCs were activated prior to infection by phytohemeagglutinin A (10 ug/ml) and interleukin-2 (10 U/ml) for 3 days.

Variants of HIV-1 LAI clone, Vpr-FS and Vpr-FS-StuI, deficient in vpr protein were generated by inserting polylinkers of 16 (Vpr-FS-StuI) or 20 (Vpr-FS) nucleotides at position 980 of the gene (details are available upon request). To generate stocks of infectious virus, 293T cells were transiently transfected with each proviral clone using TransIT reagent (Mirus, Madison, WI) and the produced virus was filtered through 0.22 µm filter (Corning) to remove cell debris. To generate 1∶1 mixture of Vpr-FS and Vpr-FS-StuI clones, small aliquots of the viruses were mixed in several ratios by volume and used to infect C8166 cells at multiplicity of infection (MOI) ∼0.1. Cell-free virus was collected three days later and used to measure the ratio of Vpr-FS to Vpr-FS-StuI in each mix (described below). The results were used to mix the two viruses at a 1∶1 infectivity ratio. The 50 µL aliquots of the mixture were stored at −80°C. Two independent mixtures were created and used for all of the experiments described here. Multiple measurements showed that Vpr-FS-StuI was found in the first and second mixture at 51.00% and 49.68% frequency, respectively. Both mixtures are referred to as “1∶1 mixture” or “50% mixture” throughout the text, but the actual frequencies were used in calculations of the variance.

Viral replication in cultures of C8166 and CEMx174 cells was monitored by an increase in SEAP activity in culture medium using PhosphaLight detection kit according to the manufacturer's instructions (Applied Biosystems, Foster City, CA). Viral replication in PBMCs was monitored by the amount of p24 antigen in cell-free media using a p24 detection kit (NCI-Frederick) and QuantaBlu Fluorogenic Peroxidase Substrate kit (Pierce) according to manufacturers' instructions.

### Viral Titers

Tissue culture infectious dose of 50% (TCID50) was measured in every experiment by the standard approach of limiting virus dilutions and counting the number of infection-negative wells in each dilution. Rows of cultures that contained virus-negative wells were maintained for 21–28 days to ensure detection of all infected wells. To calculate the number of infected cells at each dilution, TCID50 units were converted into infectious particles per milliliter by multiplying TCID50 by 0.7 (to account for the fact that Poisson distribution predicts 50% negative outcomes at ∼0.7 mean) and adjusting for the dilution factor.

In some cases, an infectious center assay was used to measure the number of infected cells in a well after infection. Cells were washed to remove any non-bound virus, serially diluted and mixed with 4×10^5^ non-infected cells in 6 replicates for each dilution. The total number of infected cells in the original cell population was calculated similar to TCID50 calculation.

### GeneScan Assay

RT-PCR-based GeneScan assay was used to measure the proportions of Vpr-FS and Vpr-FS-StuI clones in cultures as described previously [28], but with a different set of primers. The primers for PCR were designed to flank the region in the vpr gene containing insertions. The primers were 705-vpr-F1 (5′-GCCACACAATGAATGGACACTAGAGC-3′) and 710-vpr-R4 (5′-6-FAM-ATTATGGCTTCCACTCCTGCCCAAGT-3′). Briefly, virus-containing media was lysed by addition of 0.04% Triton X-100 and subjected to RT-PCR using OneStep RT-PCR kit (Qiagen, Valencia, CA). The RT step was performed at 50°C for 30 min followed by an RT-inactivation step (95°C for 15 minutes) and two-step PCR amplification (1 minute at 58°C and 15 seconds at 95°C) for 25 cycles. The PCR product was diluted with water 5–100-fold to get the fluorescent signal into the linear range of the machine, ran on Applied Biosystems sequencing machines, and the data was analyzed with free PeakScanner 1.0 software (Applied Biosystems). Due to different insertion lengths, products from Vpr-FS and Vpr-FS-StuI have different length and appear as distinct peaks. The area of each peak is calculated by the PeakScanner and is proportional to the relative amount of each PCR product.

### Using Allele Frequency Variance to Measure Genetic Drift

In an ideal population, the expected variation in neutral allele frequency, V, for a single generation depends on the initial allele frequency *p* and the population size N:
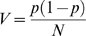



In an ideal population that changes in size over time, the expected variance of a neutral allele frequency at generation *n* is:
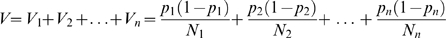



Because HIV populations in culture are growing exponentially, N_1_≪N_2_≪…≪N_n_. Therefore, the majority of the variance is contributed by the first generation and
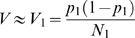



To measure the actual variance in allele frequency we infected multiple (12 to 24) cultures of cells with 1∶1 mixture of viruses Vpr-FS and Vpr-FS-StuI. The virus was allowed to spread through the culture for 5–12 days until a majority of the cells were infected. At that point, cell-free virus was collected and the proportion of Vpr-FS-StuI virus was determined by the GeneScan assay. The variance in Vpr-FS-StuI frequency was calculated as 
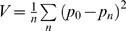
, where n is the number of cultures, *p*_0_ is the frequency of Vpr-FS-StuI in the original 1∶1 virus mixture and *p_n_* is the frequency of Vpr-FS-StuI in culture *n* at the end of experiment.

Variability within GeneScan assay itself also contributed to the observed variance in the Vpr-FS-StuI frequency. To account for that, we measured the assay variation by performing multiple assay replicates on a single randomly chosen sample. Assay variance was calculated as 
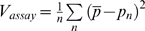
, where *p_n_* is the frequency of Vpr-FS-StuI in replicate n and 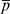 is the average frequency in all replicates. This assay variance was subtracted from the total observed variance to obtain true experimental variance.

### Statistical Methods for Comparison of Variances

To evaluate differences in observed variation between different experimental conditions, variance functions in combination with generalized linear models were used to test for differences in fold-change between the variance of each experimental condition in relation to the variance of the C8166 experiment [29]. The use of variance functions and generalized linear models allowed us to analyze the data from all experiments simultaneously, and to adjust for contributions to the overall observed variance due to differential population sizes. In addition, any correlation within data from the same experimental replicate is accounted for. This analysis provides estimates and 95% confidence intervals for the fold-difference in variation, adjusted for different population sizes within each experimental condition in relation to the C8166 experimental condition. Any experimental condition where the confidence interval does not contain the value of one has statistically significantly different variance than the C8166 experimental condition. In addition, the same test was conducted comparing the variance from the synchronized experiment to the single cycle experiment.
